# A Food-Grade Enzyme Preparation with Modest Gluten Detoxification Properties

**DOI:** 10.1371/journal.pone.0006313

**Published:** 2009-07-21

**Authors:** Jennifer Ehren, Belen Morón, Edith Martin, Michael T. Bethune, Gary M. Gray, Chaitan Khosla

**Affiliations:** 1 Department of Chemical Engineering, Stanford University, Stanford, California, United States of America; 2 Department of Chemistry, Stanford University, Stanford, California, United States of America; 3 Department of Biochemistry, Stanford University, Stanford, California, United States of America; 4 Department of Medicine, Stanford University, Stanford, California, United States of America; Cairo University, Egypt

## Abstract

**Background and Aims:**

Celiac sprue is a life-long disease characterized by an intestinal inflammatory response to dietary gluten. A gluten-free diet is an effective treatment for most patients, but accidental ingestion of gluten is common, leading to incomplete recovery or relapse. Food-grade proteases capable of detoxifying moderate quantities of dietary gluten could mitigate this problem.

**Methods:**

We evaluated the gluten detoxification properties of two food-grade enzymes, aspergillopepsin (ASP) from *Aspergillus niger* and dipeptidyl peptidase IV (DPPIV) from *Aspergillus oryzae*. The ability of each enzyme to hydrolyze gluten was tested against synthetic gluten peptides, a recombinant gluten protein, and simulated gastric digests of whole gluten and whole-wheat bread. Reaction products were analyzed by mass spectrometry, HPLC, ELISA with a monoclonal antibody that recognizes an immunodominant gluten epitope, and a T cell proliferation assay.

**Results:**

ASP markedly enhanced gluten digestion relative to pepsin, and cleaved recombinant α2-gliadin at multiple sites in a non-specific manner. When used alone, neither ASP nor DPPIV efficiently cleaved synthetic immunotoxic gluten peptides. This lack of specificity for gluten was especially evident in the presence of casein, a competing dietary protein. However, supplementation of ASP with DPPIV enabled detoxification of moderate amounts of gluten in the presence of excess casein and in whole-wheat bread. ASP was also effective at enhancing the gluten-detoxifying efficacy of cysteine endoprotease EP-B2 under simulated gastric conditions.

**Conclusions:**

Clinical studies are warranted to evaluate whether a fixed dose ratio combination of ASP and DPPIV can provide near-term relief for celiac patients suffering from inadvertent gluten exposure. Due to its markedly greater hydrolytic activity against gluten than endogenous pepsin, food-grade ASP may also augment the activity of therapeutically relevant doses of glutenases such as EP-B2 and certain prolyl endopeptidases.

## Introduction

Celiac sprue is an inheritable, life-long disease that is characterized by an inflammatory reaction to dietary gluten in the human small intestine. The hallmark of the disease is a characteristic flattening of intestinal villi along with crypt hypertrophy. As a consequence, there is tremendous loss of surface area and malabsorption of nutrients, vitamins and minerals. If untreated, celiac sprue is associated with complications such as anemia, bone diseases, infertility, neurological problems, cancer and other complications due to persistent inflammation and micronutrient deficiencies. Screening studies predict that approximately 1% of the United States' population has the disease, yet only ca. 10% of affected individuals have been diagnosed thus far [1]. At present, the only suitable treatment is strict, life-long exclusion of gluten from the patient's diet. Although a large fraction of patients who attempt to follow such a diet still exhibit signs or symptoms of active disease [2]–[5], there is no available supplementary therapy for such conditions.

A potential cost-effective solution to the aforementioned problem is an oral protease or protease mixture that is composed solely of commercially available food-grade enzymes. In this study we have evaluated the gluten detoxification properties of one such enzyme cocktail. It includes at least two proteases, aspergillopepsin (ASP) from *Aspergillus niger* and dipeptidyl peptidase IV (DPPIV) from *Aspergillus oryzae*, both of which are widely used in the foods and feeds, food processing and dietary supplement industries. Our data suggest that, whereas neither enzyme preparation alone is able to detoxify gluten under simulated gastric conditions, a defined dose ratio of the two enzyme preparations is able to detoxify moderate quantities of dietary gluten. Clinical studies are therefore warranted to evaluate the activity of this food-grade enzyme preparation in celiac sprue patients with an inadequate response to a gluten-free diet. Our results also show that, under gastric conditions, ASP augments the efficacy of gluten-detoxifying endoproteases such as the glutamine specific EP-B2 from barley [6].

## Results

### Identity

The Materials Safety Data Sheet of the commercial ASP powder from Bio-Cat Inc. designates this enzyme as aspergillopepsin from *A. niger* (EC 3.4.23.18). N-terminal sequencing of the major 41 kD protein in this powder yielded an N-terminal sequence of –K-SAVTT, consistent with its identity as aspergillopepsin A precursor (a.k.a., aspergillopepsin I) having a molecular weight of 41 kD. Mass mapping of tryptic fragments further verified its identity. Pro-aspergillopepsin A is believed to spontaneously self-activate into the mature enzyme under acidic conditions [7].

The Materials Safety Data Sheet of the commercial Peptidase P powder from Bio-Cat Inc. designates this enzyme as dipeptidyl peptidase IV from *A. oryzae* (EC 3.4.21.63). Although DPPIV activity could be verified by the chromogenic substrate Gly-Pro-pNA, SDS-PAGE revealed that the enzyme preparation was a complex mixture of proteins that lacked a prominent band with MW higher than 75 kD, as might be expected for this DPPIV [8]. We therefore used an alternate strategy to verify the identity of this serine protease in the commercial enzyme preparation. An inhibition assay was performed with various concentrations of known DPPIV inhibitor, Boc-L-Prolinal. At a concentration of 9 mM inhibitor in the reaction volume, 100% inhibition of DPPIV (0.1 mg/ml) activity was observed.

### Activity and stability of commercial ASP and DPPIV preparations

The total protein content and specific activity of food-grade ASP and DPPIV evaluated in this study is summarized in Table 1. Periodic analysis in our laboratories demonstrated that both enzyme powders were stable for at least 6 months at room temperature.

**Table 1:** Protein content and specific activity of aspergillopepsin (ASP) and dipeptidyl peptidase IV (DPPIV) preparations used in this study.

	ASP	DPPIV
Total protein (t = 0)	14±0.6%	16±0.6%
Total protein (t = 6 mo)	13±0.4%	15±0.7%
Specific activity (t = 0)	15.2±0.2 units/µg	2.3±0.1 units/g
Specific activity (t = 6 mo)	13.4±0.4 units/µg	2.7±0.1 units/g

Each measurement is an average of four enzyme samples, stored and analyzed independently.

### Hydrolysis of gluten-derived proteins and peptides by ASP and DPPIV

**Figure 1:**
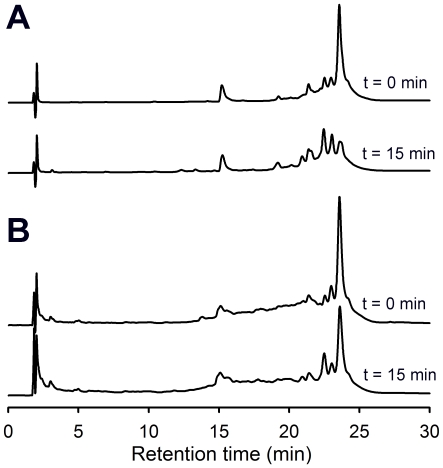
Digestion of the proteolytically resistant 33-mer peptide from α2-gliadin with aspergillopepsin (ASP). (A) Reverse-phase HPLC traces of the 33-mer peptide incubated with ASP in a 20∶1 (w/w) peptide:enzyme ratio at pH 4.5. (B) Reverse-phase HPLC traces of the 33-mer peptide mixed with gluten digested by pepsin, and incubated with ASP in a 20∶80∶1 (w/w/w) peptide:gluten:enzyme ratio at pH 4.5. The ordinate scale is identical in all traces. The peak at 15 min corresponds to an internal standard (TAME).

It is well established that mammalian pepsin exerts only minimal proteolytic action on dietary gluten. Our search for food-grade gluten detoxifying enzymes therefore led us to aspergillopepsin as a potentially more potent analog of mammalian pepsin. We first wished to investigate the specificity of ASP towards recombinant gluten proteins and synthetic gluten peptides. Available literature suggests that ASP cleaves proteins relatively non-specifically. For example, ASP cleaves ribonuclease A at Tyr-X, Phe-X, His-X, Asn-X, Asp-X, Gln-X and Glu-X bonds [9]. LC-MS analysis revealed that ASP cleaved the peptide VQWPQQQPVPQPHQPF from γ-gliadin after Q5, P8 and V9 residues, and also cleaved the peptide PFSQQQQPV from glutenin after Q4, Q5 and Q6 residues. In the absence of other protein substrates, ASP cleaved the 33-residue peptide LQLQPFPQPQLPYPQPQLPYPQPQLPYPQPQPF from α2-gliadin (Figure 1A), as well as its truncated 28-residue derivative PFPQPQLPYPQPQLPYPQPQLPYPQPQP (data not shown). However, in the presence of the more complex substrate whole gluten, ASP exhibited minimal activity toward the 33-mer (Figure 1B), indicating that the enzyme has low specificity for this immunotoxic epitope. Other immunotoxic gluten peptides, such as the 26-mer FLQPQQPFPQQPQQPYPQQPQQPFPQ from γ5-gliadin [10] and the innate immune peptide LGQQQPFPPQQPYPQPQPF [11], were similarly resistant to cleavage by ASP (data not shown).

**Figure 2:**
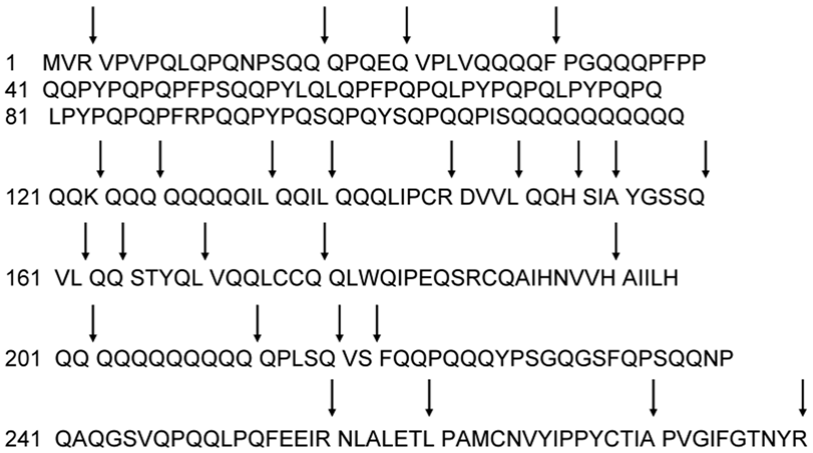
Proteolytic specificity of aspergillopepsin (ASP) for α2-gliadin. The purified, recombinant protein substrate was incubated for 15 min with food-grade ASP in a 50∶1 (w/w) substrate:enzyme ratio at pH 4.5. Cleavage sites were identified by LC/MS/MS, and are indicated by arrows.

Although ASP is not specific for immunotoxic gluten epitopes, it may potentiate gluten detoxification by cleaving larger proteins into short peptides that are accessible substrates for more specific endopeptidases and exopeptidases [12], [13]. To more thoroughly investigate the potential of ASP for gluten detoxification, full-length recombinant α2-gliadin protein was treated with ASP for 15 min (substrate: enzyme ratio of 50∶1, pH 4.5), and the resulting peptide mixture was analyzed by LC-MS-MS. The data were searched against the SwissProt database, from which α2-gliadin was correctly identified as the best hit. Those peptides with ion scores >30 were used to construct a cleavage map. Our analysis, summarized in Figure 2, revealed that ASP cleaved α2-gliadin at a wide range of sites, including H-X (X = S,A), Q-X (X = Q,V,S,E), R-X (X = V,N), A-X (X = Y,P), K-Q, L-X (X = Q,V,P), F-X (X = P,E), P = X (X = V,Q), T-X (X = I,N), Y-R, and S-X (X = F,Q) bonds.

**Figure 3:**
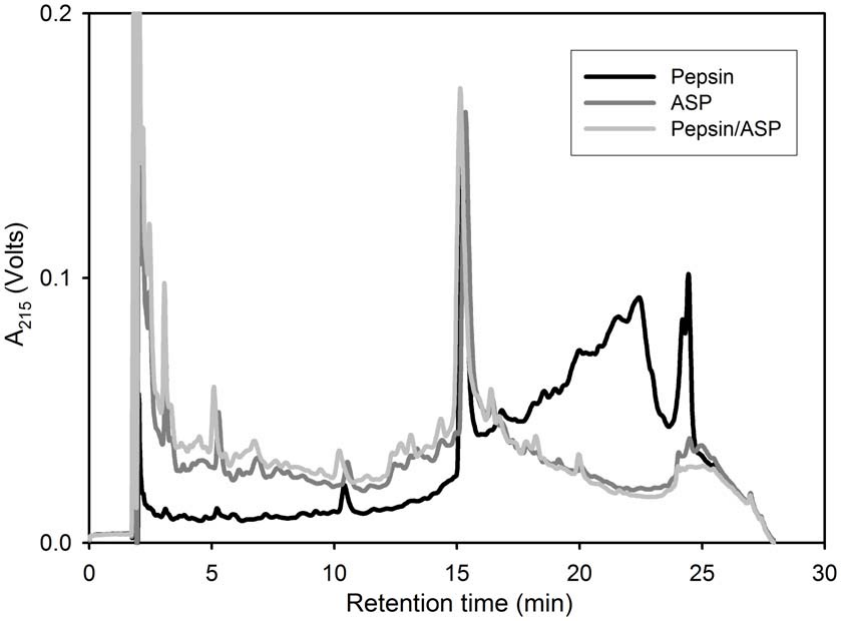
Proteolysis of whole gluten by aspergillopepsin (ASP) under simulated gastric conditions. Whole gluten powder (15 mg/ml) was incubated with either 0.35 mg/ml ASP, 0.6 mg/ml pepsin, or both enzymes at 37°C for 60 min at pH 2. The resulting product mixture was analyzed by reverse-phase HPLC, as described in the text. The breakdown of longer gluten peptides into shorter ones is generally indicated by a decrease in absorbance at higher retention times and a concomitant increase in absorbance at lower retention times. The peak at 15 min corresponds to an internal standard (TAME).

**Figure 4:**
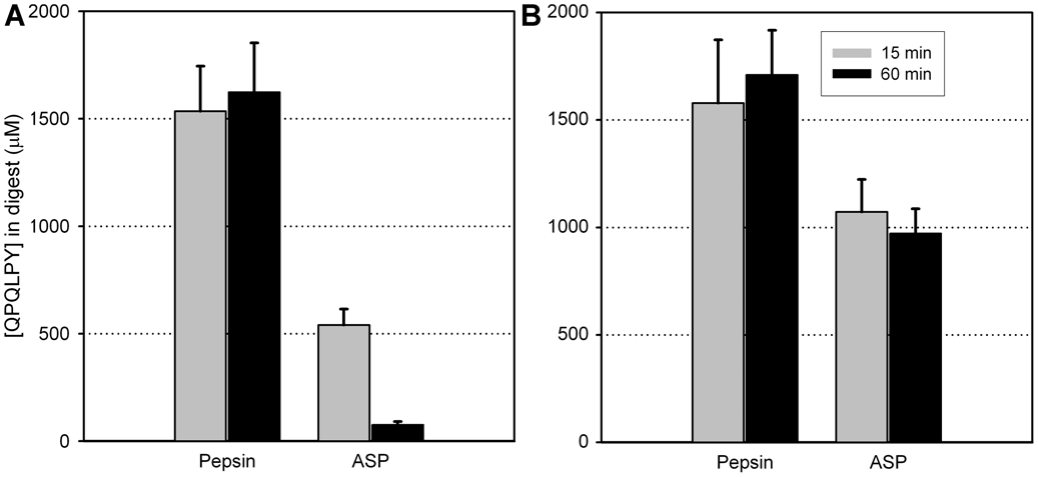
Detoxification of gluten by aspergillopepsin (ASP) under conditions simulating gastric digestion of a meal. (A) Whole gluten powder (15 mg/ml) or (B) whole gluten powder (15 mg/ml) mixed with casein (50 mg/ml) was incubated with either 0.6 mg/ml pepsin or 0.35 mg/ml ASP at 37°C for 15 or 60 min in 0.03 M HCl. Gliadin peptides present in the digests were analyzed by competitive ELISA using the G12 monoclonal antibody, which is specific for the immunotoxic gliadin epitope QPQLPY.

Based on these findings with structurally defined substrates, we hypothesized that, although ASP was considerably more effective than mammalian pepsin at cleaving gluten into short peptides, it would have limited ability to detoxify gluten in the context of a complex meal. To test this hypothesis, we compared the activity of pepsin versus ASP against 15 mg/ml whole gluten or 15 mg/ml gluten mixed with 50 mg/ml casein under simulated gastric conditions (0.03 M HCl). The markedly superior activity of ASP against whole gluten is illustrated in Figure 3. However, as seen in Figure 4, in the presence of a more abundant dietary protein such as casein, ASP-catalyzed gluten detoxification is extremely retarded. These results suggested that ASP mediated gluten detoxification in the context of a complex meal would require a second, complementary enzyme.

**Figure 5:**
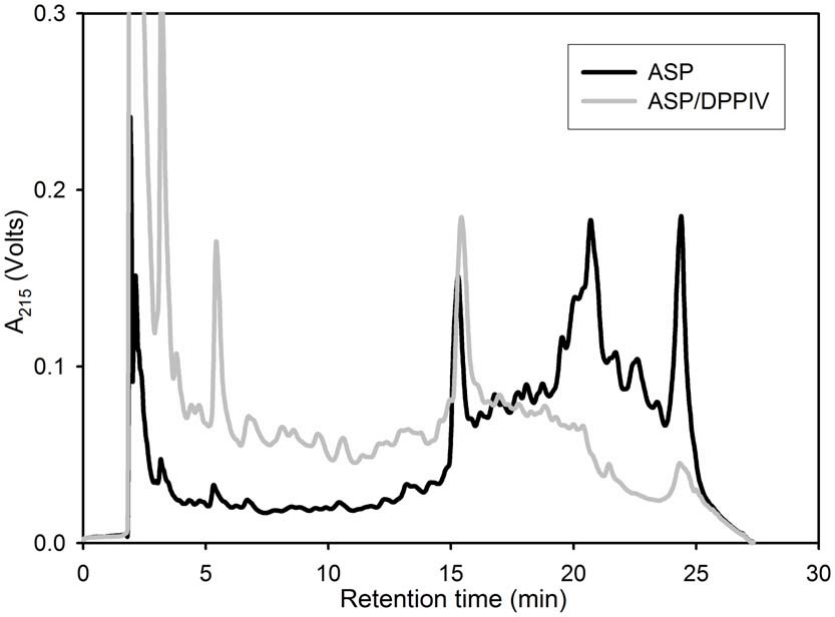
Effect of a food-grade dipeptidyl peptidase IV (DPPIV) preparation on gluten proteolysis by aspergillopepsin (ASP). Whole gluten powder (50 mg/ml) was incubated for 60 min at 37°C with 0.35 mg/ml ASP in the presence or absence of 0.7 mg/ml DPPIV. Because this enzyme preparation is inactive at pH <4, the reaction was conducted in 0.03 M HCl in the presence of 1.5 mg/ml CaCO_3_ to simulate addition of a standard over-the-counter antacid/calcium supplement. The pH of the resulting mixture varies between 4–5 for the duration of the reaction. The peak at 15 min corresponds to an internal standard (TAME).

Preliminary studies with a variety of other over-the-counter enzymes led us to conduct more detailed evaluations of peptidase P from *Aspergillus oryzae*. This commercial enzyme preparation contains the exopeptidase DPPIV, and is also widely used as a debittering agent in foods and dietary supplements due to its specificity for peptides with N-terminal X-P- sequences. Although the enzyme is completely inactive against gluten at pH <4, at higher pH values it markedly enhances the extent to which ASP hydrolyzes gluten (Figure 5). In earlier studies we observed that fungal DPPIV was unable to detoxify immunotoxic gluten peptides when added as a lone supplement to gastrointestinal proteases [13]. We reconfirmed this lack of utility of DPPIV as a single agent by incubating commercial peptidase P with synthetic 33-mer from α2-gliadin or the 26-mer from γ5-gliadin or the innate immune peptide p31–49. In each case, minimal-to-no peptide hydrolysis occurred, with the only new products being slightly truncated analogs of the starting material (data not shown). Thus, to the extent a glutenase cocktail could be formulated by combining ASP and DPPIV, it would presumably detoxify gluten in the stomach via the successive action of ASP on intact gluten proteins followed by DPPIV on the peptide generated by ASP. This hypothesis was tested in a variety of simulated gastric studies outlined below. Due to the pH constraints of DPPIV, this enzyme cocktail required supplementation of the gastric digests with antacid, a necessity likely to carry over to future studies *in vivo*.

### Simulated gastric digestion of whole gluten and whole wheat bread

**Figure 6:**
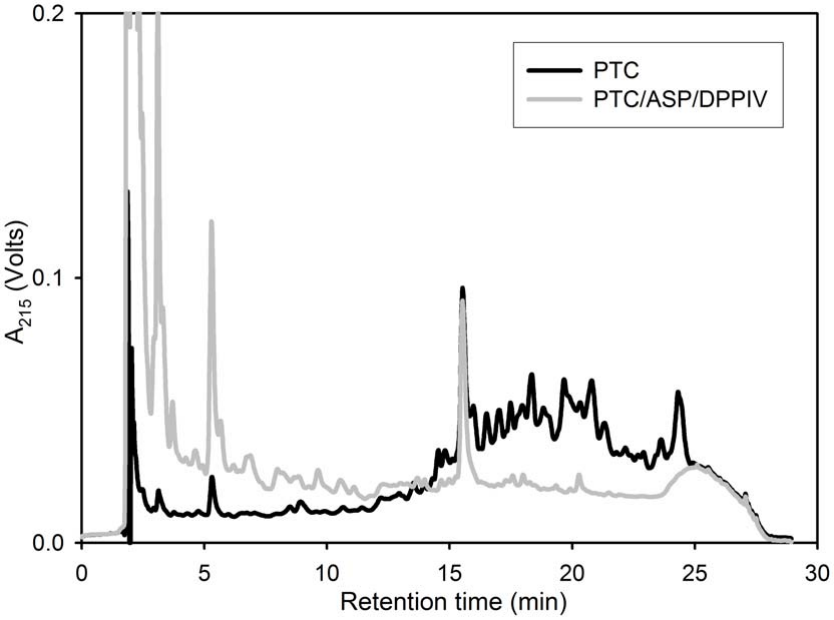
Effect of aspergillopepsin (ASP) and dipeptidyl peptidase IV (DPPIV) on the proteolysis of baked gluten in whole wheat bread under simulated gastric and duodenal conditions. The reverse phase HPLC traces show the residual peptide content after whole wheat bread was incubated with pepsin (0.6 mg/ml) under simulated gastric conditions, followed by trypsin (0.375 mg/ml) and chymotrypsin (0.375 mg/ml) under simulated duodenal conditions (PTC) or PTC+ASP (0.35 mg/ml)+DPPIV (0.7 mg/ml). The peak at 15 min corresponds to an internal standard (TAME). For experimental details, see text.

We first wished to verify the proteolytic action of ASP and DPPIV on baked gluten in whole wheat bread. For this purpose digestions and sample analyses were performed under simulated gastric conditions developed earlier for bread [6]. Notably, at the end of the gastric phase of digestion, samples were neutralized and treated with trypsin and chymotrypsin to mimic the duodenal phase of protein digestion. The reason for this additional step was to be able to reliably compare the effect of ASP and DPPIV relative to pepsin alone via reverse phase HPLC, as pepsin alone has negligible proteolytic action on baked gluten [6]. As shown in Figure 6, addition of ASP+DPPIV to pepsin leads to a marked enhancement of gluten digestion under simulated gastric conditions.

**Figure 7:**
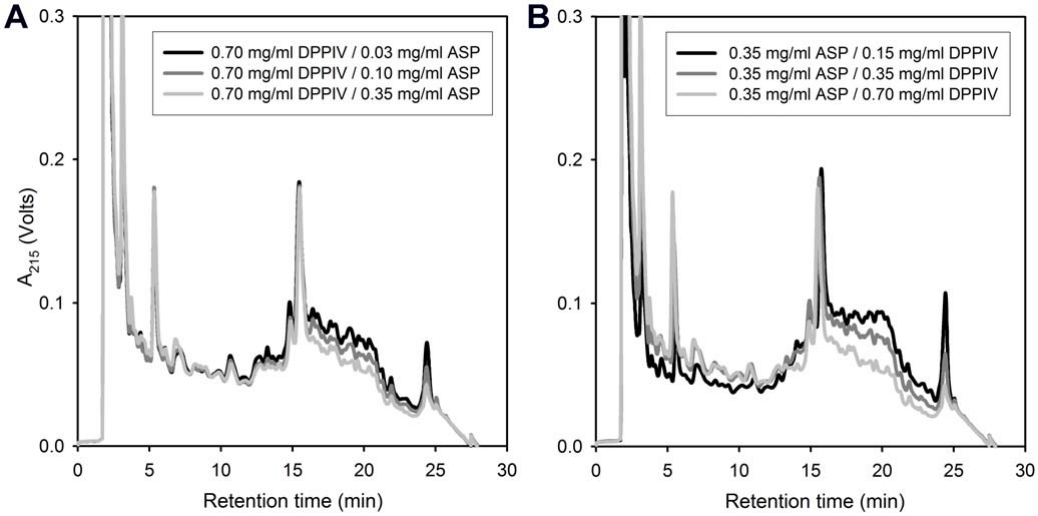
Dose dependence of gluten proteolysis on aspergillopepsin (ASP) and dipeptidyl peptidase IV (DPPIV). Whole gluten powder (50 mg/ml) was incubated for 60 min at 37°C with 0.7 mg/ml DPPIV and variable ASP concentration (A) or 0.35 mg/ml ASP and variable DPPIV concentration (B). The reactions were conducted in 0.03 M HCl in the presence of 3 mg/ml CaCO_3_. The peak at 15 min corresponds to an internal standard (TAME).

To assess the dependence of gluten proteolysis on ASP and DPPIV dose, we digested 50 mg/ml gluten in the presence of 0.03 M HCl and 3 mg/ml CaCO_3_ by varying the concentration of one enzyme while keeping the other constant. Representative data is shown in Figure 7. Whereas the activity of ASP on such a high gluten concentration showed a relatively small increase in activity over a 10-fold range of enzyme (Figure 7A), a progressive increase in DPPIV activity is seen up to the highest achievable concentration (0.7 mg/ml) of this enzyme preparation (Figure 7B). Based on these studies, we estimated that ASP and DPPIV protein concentrations of 0.35 mg/ml and 0.7 mg/ml would be practically achievable targets in the post-prandial stomach (corresponding to oral doses of 250 mg ASP and 400 mg DPPIV).

**Figure 8:**
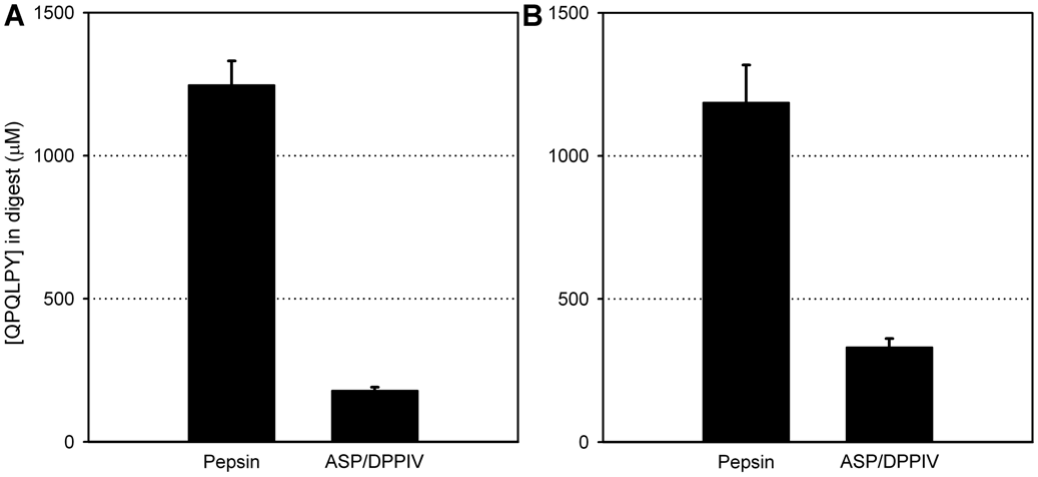
Gluten detoxifying activity of aspergillopepsin (ASP) and dipeptidyl peptidase IV (DPPIV) under conditions simulating gastric digestion of a meal. (A) Whole gluten powder (15 mg/ml) or (B) whole gluten powder (15 mg/ml) mixed with casein (50 mg/ml) was incubated with either 0.6 mg/ml pepsin or 0.35 mg/ml ASP and 0.7 mg/ml DPPIV at 37°C for 60 min. The digestions were initiated in 0.03 M HCl and 3 mg/ml CaCO_3_, and every 5 min additional HCl was added. Gliadin peptides present in the digests were analyzed by competitive ELISA using the G12 monoclonal antibody, which is specific for the immunotoxic gliadin epitope QPQLPY.

Additionally, to assess the potential of the above ASP+DPPIV cocktail for detoxifying gluten under conditions that more accurately mimic the continuously changing environment of the post-prandial stomach, gluten (alone or in combination with casein) was digested with either pepsin or ASP+DPPIV. This experiment was started by adding gluten (or gluten plus casein), an appropriate quantity of each enzyme and 3 mg/ml CaCO_3_ to 0.03 M HCl to simulate the entry of food and a physiological quantity of antacid in the empty stomach. At this point the pH of the mixture was 3 (in the case of gluten alone) or 4 (in the case of gluten+casein). Every 5 min thereafter, an additional 0.01 M equivalent of concentrated HCl was added in order to simulate the periodic squirting of acid into the fed stomach. After 1 hr, the pH was 1 (in the case of gluten alone) or 2.5 (in the case of gluten+casein). At this time-point, samples were withdrawn from each reaction mixture and analyzed via a competitive ELISA assay that is specific for a representative immunotoxic epitope. Treatment of gluten or gluten plus casein with ASP+DPPIV reduced the epitope concentration by 7- or 4-fold, respectively, compared to the samples treated with pepsin only (Figure 8).

**Figure 9:**
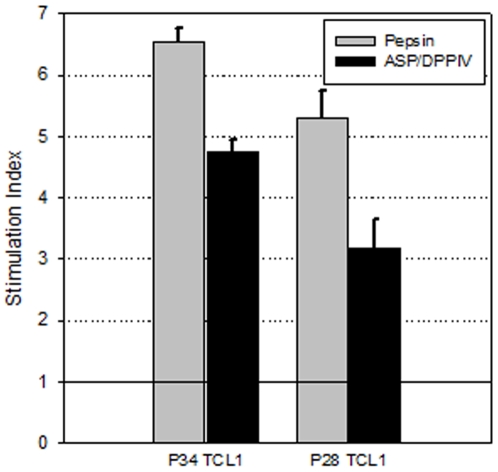
Stimulation of T-cell lines derived from biopsies of celiac sprue patients with gluten digested by aspergillopepsin (ASP) and dipeptidyl peptidase IV (DPPIV). Whole gluten powder (15 mg/ml) mixed with casein (50 mg/ml) was incubated with either 0.6 mg/ml pepsin or 0.35 mg/ml ASP and 0.7 mg/ml DPPIV. The digestions were initiated in 0.03 M HCl and 3 mg/ml CaCO_3_, and every 5 min additional HCl was added. After digestion for 60 min at 37°C, the samples were further treated with 0.375 mg/ml trypsin and 0.375 mg/ml chymotrypsin for 30 min at pH 6.0. The immunotoxic peptide content in the water-soluble fraction of the digests was measured by a T-cell proliferation assay. A stimulation index of 1 indicates background levels of T-cell proliferation and is denoted with a horizontal line. The name of the individual T-cell lines is indicated in the graph.

Finally, to confirm that the reduction in QPQLPY epitope measured by competitive ELISA (Figure 8) correlates with reduced ability of the ASP/DPPIV digests to stimulate gluten-responsive T-cell lines, gluten and casein digests were analyzed by both the G12 competitive ELISA and a standard T-cell proliferation assay. For this comparison only the water-soluble fractions of the digests were analyzed as the T-cell proliferation assay, although a more pathogenically relevant measure of immunotoxicity, is not amenable to measuring the latent immunotoxic gliadin epitopes of ethanol extracts. The samples were therefore digested under simulated gastric conditions as described for Figure 8, followed by trypsin and chymotrypsin treatment for 30 min. This last step was added in order to increase the amount of gluten peptides present in the water-soluble fraction, as pepsin has poor proteolytic activity on dietary gluten. As shown in Figure 9, the combination of ASP and DPPIV was able to reduce the amount of T-cell stimulatory peptides in the digests, compared to the samples digested with pepsin, trypsin and chymotrypsin. These results are consistent with the QPQLPY concentration in the same digests measured by G12 competitive ELISA, in which QPQLPY amount was 192.5±12.4 µM in pepsin, trypsin and chymotrypsin digests, and 64.9±5.5 µM in the samples where ASP and DPPIV were added.

### Comparison with other glutenases

**Figure 10:**
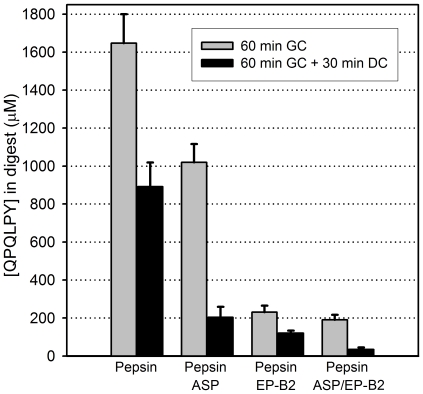
Detoxification of gluten by aspergillopepsin (ASP) and EP-B2 under conditions simulating digestion of a meal. Whole gluten powder (15 mg/ml) and casein (50 mg/ml) were digested with 0.6 mg/ml pepsin for 60 min at pH 4 (gastric conditions, GC), followed by treatment with 0.375 mg/ml trypsin and 0.375 mg/ml chymotrypsin for 30 min at pH 6.0 (duodenal conditions, DC). Where indicated, 0.35 mg/ml ASP, and/or 0.375 mg/ml proEP-B2 were added at the beginning of the gastric digestion phase. Gliadin peptides present in the digests were analyzed by competitive ELISA using the G12 monoclonal antibody, which is specific for the immunotoxic gliadin epitope QPQLPY.

As discussed above, an attractive feature of aspergillopepsin is that, unlike mammalian pepsin, aspergillopepsin is able to extensively hydrolyze dietary gluten into short peptides. This finding suggests that ASP should be able to complement the glutenase activities of other promising enzymes such as the glutamine-specific cysteine endoprotease EP-B2 from barley [14], the proline-specific prolyl endopeptidase AN PEP from *A. niger* [15], and the combination product composed of EP-B2 and prolyl endopeptidase SC PEP from *S. capsulata* [6]. To test this hypothesis, we assessed the proteolytic activity of the proenzyme form of EP-B2 in the presence or absence of ASP on a mixture of gluten and casein. Whereas EP-B2 is highly effective at cleaving the 33-mer peptide from α2-gliadin, the toxic heptameric epitope QLPYPQP in wheat bread digests is not hydrolyzed completely by EP-B2 alone [20]. As seen in Figure 10, a combination of both EP-B2 and ASP led to a further reduction in the concentration of the related epitope QPQLPY than either enzyme alone. With respect to pepsin alone, 33-mer content in these digests measured by triple quadrupole LC-MS was reduced 22% by ASP and was not detectable (corresponding to ≥98% reduction) by EP-B2 or by EP-B2 plus ASP.

## Discussion

There is a cogent need for some form of near-term supportive therapy for celiac sprue, because dietary gluten exclusion is often less than complete. Patients on a restricted diet frequently suffer nutrient and mineral malabsorption, resulting in deficiencies of iron or calcium, and consequently in anemia and osteoporosis, respectively. This has motivated us to evaluate commercially available food-grade enzymes for their ability to detoxify low-to-moderate doses of gluten. By way of calibration, one slice of wheat bread contains ca. 3–4 g gluten, whereas the threshold of “safe” gluten for celiac patients is estimated to be in the 10–100 mg range [16]. In most cases, inadvertent gluten exposure results from consumption of 10–1000 mg of “invisible” gluten found in commonplace food ingredients such as sauces, salad dressings, food starches, thickeners malt extract and other flavoring agents, or sometimes simply through cross-contamination during food preparation. Therefore, we sought to devise an oral enzyme cocktail based upon existing over-the-counter products that may be able to detoxify up to 1 g gluten while the food is still in the stomach.

Aspergillopepsin, unlike mammalian pepsin, is able to extensively hydrolyze dietary gluten into short peptides. However, in contrast to glutenases such as prolyl endopeptidases or barley EP-B2, which have high specificity for immunotoxic Pro- and Gln-rich gluten peptides such as the 33-mer from α2-gliadin, ASP lacks specificity for these peptides. By virtue of its relatively low sequence specificity, it can hydrolyze these peptides, but the presence of competing substrates slows this hydrolysis considerably. The extensive exoproteolytic action of a second enzyme such as the fungal DPPIV accelerates clearance of short peptides, thereby facilitating the access of longer gluten peptides to the ASP active site and their eventual detoxification [13]. In short, our *in vitro* studies suggest that detoxification of a low-dose of gluten can be achieved using ASP and DPPIV. Since these enzymes have already been proven safe for human consumption, this dual-therapy holds promise for the near-term relief of the inflammatory intestinal response of celiac patients who suffer from inadvertent gluten exposure. In addition, ASP may be added to more potent and specific glutenases such as EP-B2 [6] and certain microbial prolyl endopeptidases ([6], [17]) to further enhance their therapeutic potency. Controlled clinical studies of these food-grade enzymes are therefore warranted.

## Materials and Methods

### Materials

Food-grade aspergillopepsin (ASP) from *Aspergillus niger* and dipeptidyl peptidase IV (DPPIV) from *Aspergillus oryzae* enzymes were supplied in powder form by Bio-Cat, Inc (Troy, VA). Whole gluten was from Bob's Red Mill (Milwaukie OR), and whole wheat bread was from Alvarado St. Bakery (Rohnert Park, CA). Pepsin was obtained from American Laboratories (Omaha, NE). Trypsin (from bovine pancreas, T4665), α-chymotrypsin (type II from bovine pancreas, C4129), bovine hemoglobin (H-2625) and casein (from bovine milk, C7078) were from Sigma (St. Louis, MO). The substrate for assaying DPPIV activity (Gly-Pro-p-nitroanilide) was from Bachem (Torrance, CA).

### Protein and peptide production

Gluten peptides were synthesized on solid-phase using Boc/HBTU chemistry, purified by reverse phase HPLC, and lyophilized as described [18]. Peptides were resuspended in 50 mM sodium phosphate, pH 7.0+0.02% NaN_3_ prior to use. Recombinant α2-gliadin was expressed heterologously in *E. coli* and purified as described [19].

### Identity

The identity of aspergillopepsin was confirmed via N-terminal sequence analysis and mass spectrometry of a trypsin digest of the major protein observed at 41 kD by SDS-PAGE. Due to its low abundance in the commercial enzyme powder from *A. oryzae*, the identity of the DPPIV protein could not be unequivocally established by protein sequencing. However, to confirm the presence of DPPIV in this powder, we performed two assays. First, a standard chromogenic assay for DPPIV was performed using Gly-Pro-p-nitroanilide as a substrate. Second, an inhibition assay was performed with a known DPPIV inhibitor, Boc-L-Prolinal.

### Specificity

The specificity of ASP was assessed by LC/MS/MS analysis of α2-gliadin digested with the commercial enzyme powder from *A. niger*, as previously described [14]. In brief, digestions were performed at a substrate: enzyme ratio of 50∶1 at pH 4.5 for 15 minutes. Additional analysis was conducted with the following immunogenic peptides: LQLQPFPQPQLPYPQPQLPYPQPQLPYPQPQPF and its truncated analog PFPQPQLPYPQPQLPYPQPQLPYPQPQP, and the innate immune peptide LGQQQPFPPQQPYPQPQPF from α-gliadin; two γ-gliadin peptides, FLQPQQPFPQQPQQPYPQQPQQPFPQ and VQWPQQQPVPQPHQPF; and a glutenin peptide PFSQQQQPV.

### Assays to measure enzyme specific activity

The protein concentration in each commercial enzyme preparation was determined by the Bradford protein assay. A standard calibration curve was generated using bovine serum albumin in the concentration range of 2–12 µg/ml.

ASP activity was measured using the spectrophotometric hemoglobin units of tyrosine (HUT) assay. The amount of tyrosine liberated as trichloroacetic acid-soluble peptides upon hemoglobin digestion was quantified by monitoring absorbance at 280 nm. In a total reaction volume of 1.5 ml, 1.3% (w/v) of bovine hemoglobin was reacted at 37°C with three separate enzyme concentrations (final concentrations of 1.7 µg/ml, 5 µg/ml, and 8 µg/ml on a total protein basis). After 10 min, the reaction was quenched using trichloroacetic acid (TCA, Sigma 490–10) added to a final concentration of 3.2% (w/v). Samples were centrifuged and the A_280_ was recorded. One HUT unit of protease activity is defined as that amount of enzyme that produces an A_280_ of 0.001 per min at pH 2.0 and 37°C, measured as TCA-soluble products using hemoglobin as a substrate (final volume = 4 ml, light path = 1 cm).

DPPIV activity was measured via a standard kinetic assay, using the chromogenic substrate Gly-Pro-p-nitroanilide dissolved in phosphate buffered saline, pH 7.4. Absorbance was monitored at 410 nm. One unit of DPPIV activity is defined as the amount of enzyme that produces 1 µmol of p-nitroaniline per min at pH 4.5 and room temperature.

### Simulated gastric and duodenal digestion of gluten

To evaluate the gluten detoxifying activity of a given enzyme or enzyme cocktail, an experimental protocol developed earlier was used to mimic the gastric digestion of either whole gluten or whole wheat bread *in vitro* [6].

To simulate duodenal digestion, we adjusted the pH to 6.0 at the end of the gastric phase. Pancreatic enzymes (trypsin and chymotrypsin) were added to yield final concentrations of 0.375 mg/ml each. Addition of elastase and carboxypeptidase A have minimal incremental effect on gluten digestion and thus were not added [6], [20]. The solution was then incubated at 37°C for up to 30 min. Samples were taken at various time-points and heat-treated as previously described.

### Reverse Phase High Performance Liquid Chromotography (HPLC)

Digested gluten or bread samples were centrifuged for 10 min at 13,400•g and filtered through a 0.2 µm low protein binding filter. The filtrate was chromatographically separated on a 4.6×150 mm reverse phase C_18_ protein and peptide column (Vydac, Hesperia, CA) using Dynamax SD-200 pumps (Varian, Palo Alto, CA) (1 ml/min), a Varian 340 UV detector set at 215 nm and a Varian Prostar 430 autosampler. Solvent A was water with 5.0% acetonitrile and 0.1% TFA. Solvent B was acetonitrile with 5.0% water and 0.1% TFA. Samples were analyzed using a gradient described previously [6].

### Competitive ELISA for immunotoxic gliadin epitopes

Relative amounts of immunotoxic gliadin epitopes in any gluten-containing sample were quantified by a competitive enzyme-linked immunoabsorbent assay (ELISA) using the horseradish peroxidase-conjugated G12 monoclonal antibody (Biomedal, Seville, Spain) [21] that is specific for the hexapeptide QPQLPY from the 33-mer peptide of α2-gliadin [19]. Residual gliadin peptides in a given sample were recovered by fully dissolving in 60% ethanol at room temperature. On day 1 of the ELISA procedure, the Maxisorp plates (Nalge Nunc, Rochester, NY) were coated with 100 µl/well of a standard gliadin solution (Sigma) (5 µg/ml, diluted in 20 mM phosphate buffer) and 100 µl of 0.02 M sodium carbonate buffer (pH 9.6), and incubated 1 h at 37°C and overnight at 4°C. On day 2, 100 µl of different dilutions (1∶500 to 1∶160,000) of each sample as well as standard solutions of the synthetic 33-mer peptide (0–10 µg/ml) were incubated for 2 hr at room temperature with 100 µl G12-HRP antibody solution (diluted 1∶10,000 in PBS containing 3% bovine serum albumin). The gliadin-coated plates were then washed twice with washing buffer (PBS containing 0.05% Tween-20, 300 µl/well), and blocked with blocking solution (washing buffer plus 5% powder milk, 300 µl/well). Antibody and antigen (standards or samples) mixes were added to the wells in duplicate (200 µl/well) and incubated for 30 min at room temperature. Plates were washed five times and exposed to 100 µl per well of HRP substrate (Sigma, St. Louis, MO). After incubation for 30 min at room temperature, color development was stopped with 1 M sulfuric acid (100 µl per well) and the absorbance was measured at 450 nm. The concentration of the QPQLPY epitope was determined using Sigma Plot 9.0 (Systat Software, Point Richmond, CA) and a 4-parameters model. Each sample was analyzed in duplicate.

### 3Q LC-MS/MS analysis of 33-mer

The content of 33-mer peptide present in gluten digests was determined by triple quadrupole LC-MS. Whole gluten (15 mg/ml) and casein (50 mg/ml) were digested with pepsin for 60 min at pH 4.0 followed by treatment with 0.375 mg/ml trypsin and 0.375 mg/ml chymotrypsin for 30 min at pH 6.0. In designated digests, pepsin was supplemented with 0.35 mg/ml ASP and/or 0.375 mg/ml EP-B2. Heat-quenched digests were centrifuged (16,100 x g) and the supernatants were diluted in an equal volume of cold acetonitrile containing 0.1% formic acid to precipitate larger proteins. Samples were vortexed, incubated for 2 h at 4°C, and centrifuged (16,100 x g) for 10 min at 4°C. Supernatants were mixed with an equal volume of 0.1% formic acid in water to dilute the acetonitrile concentration to 25%, and injected on a Micromass Quattro Premier triple quadrupole LC-MS system. The concentration in each sample was determined by comparison to a 33-mer standard curve. The limit of quantification was 2 nM.

### Gluten Deamidation

Digests were treated with 100 µg/ml recombinant human transglutaminase 2 in 200 mM MOPS, pH 7.2, and 15 mM CaCl_2_ for 2 h at 37°C. The samples were heated at >95°C for at least 5 min to inactivate the enzyme, centrifuged for 2 min at 13,000 rpm to pellet insoluble material, and frozen at −20°C until use in T cell proliferation assays.

### T-Cell Lines and ^3^H Thymidine T-Cell Proliferation Assay

T-cell proliferation assays were performed using DQ2 homozygous 9088 cells and T-cell lines P28 TCL1 and P34 TCL1 as described in Siegel et al. ([22]) except 1 µCi/well of [methyl-^3^H]-thymidine was pulsed for 15 h before harvesting the cells. Samples were diluted to protein concentrations ranging from 0.001 to 1 mg/ml. Samples were analyzed in duplicate. Stimulation index was determined as the radioactive counts per minute (CPM) in the presence of the peptide divided by the CPM in the absence of the peptide.
